# End-of-Life Perspectives and Needs for Hospice Care Among Rural Chinese Older Adults in Isolated Mountain Areas

**DOI:** 10.1093/geroni/igaf122.2314

**Published:** 2025-12-31

**Authors:** Heshuo Yu

**Affiliations:** Fudan University, Shanghai, Shanghai, China (People’s Republic)

## Abstract

Hospice care has become more prevalent in China since 2017; however, it remains largely inaccessible in isolated rural mountain areas. Discussions about death are often considered cultural taboos, and older adults in these remote regions face significant disparities in end-of-life care due to lower socioeconomic status, limited transportation, and inadequate healthcare infrastructure. Few studies have examined how this population perceives “good death” or their willingness to receive hospice care. This study explores the expectations and needs of rural Chinese older adults regarding “good death” and their perceptions of hospice care. Using a qualitative descriptive approach, we conducted semi-structured, open-ended interviews with 21 older adults and analyzed the data inductively through thematic analysis. Five key themes emerged: being accompanied by family, attaining inner peace, experiencing no physical pain, departing with dignity, and preferring to pass away at home. Cultural values deeply rooted in the Chinese context shape these perceptions. Many participants expressed a strong attachment to their home villages and a preference for in-home death, reflecting the belief that “fallen leaves return to their roots.” Fear of pain, particularly from terminal diseases like cancer, was common, and participants showed a strong willingness to use hospice care if affordable. The presence of adult children at the end of life was deemed crucial despite geographic challenges. Future research should explore cross-national perspectives on hospice care utilization and definitions of “good death” across cultures. Further study is also needed to incorporate the perceptions of other key stakeholders, including family caregivers and local healthcare providers.

